# Circulating Neopterin Is Independently Associated with Autonomic Neuropathy in Type 2 Diabetes, but Not with Other Microvascular Complications

**DOI:** 10.3390/medsci14020166

**Published:** 2026-03-26

**Authors:** Diana Nikolova, Zdravko Kamenov, Julieta Hristova, Antoaneta Trifonova Gateva

**Affiliations:** 1 Department of Internal Medicine, Aleksandrovska University Hospital, Medical University of Sofia, 1431 Sofia, Bulgaria; zkamenov@medfac.mu-sofia.bg (Z.K.); agateva@medfac.mu-sofia.bg (A.T.G.); 2 Department of Clinical Laboratory, Aleksandrovska University Hospital, Medical University of Sofia, 1431 Sofia, Bulgaria

**Keywords:** diabetic autonomic neuropathy, neopterin, biomarkers, inflammation, metabolic disorders

## Abstract

**Background**: Chronic low-grade inflammation plays a central role in the pathogenesis of type 2 diabetes (T2DM) and its complications. Neopterin, a marker of macrophage activation and Th1-mediated immune response, has been associated with cardiovascular disease and metabolic disorders. However, its relationship with diabetic autonomic neuropathy remains insufficiently investigated. **Methods**: We conducted a cross-sectional study including 129 participants (93 with T2DM and 36 with obesity without carbohydrate disturbances). Clinical, anthropometric, and biochemical assessments were performed. Cardiovascular autonomic neuropathy was evaluated using Ewing cardiovascular reflex tests and sudomotor dysfunction scoring. Neopterin concentrations were measured in serum. Correlation, ROC, and logistic regression analyses were performed. **Results:** Neopterin levels were not significantly different between T2DM and obesity groups. No differences were observed in patients with versus without peripheral neuropathy, nephropathy, or retinopathy. However, neopterin levels were significantly higher in individuals with cardiovascular autonomic neuropathy (*p* = 0.013). Neopterin correlated with cardiovascular autonomic neuropathy score, sudomotor dysfunction, fasting glucose, fasting insulin, and HOMA-IR. It showed a moderate negative monotonic correlation with eGFR (Spearman’s rho = −0.41, *p*< 0.001). In multivariable logistic regression adjusted for age, HbA1c, BMI, eGFR, and diabetes duration, each 1-SD increase in neopterin was associated with 2.67-fold higher odds of cardiovascular autonomic neuropathy (95% CI 1.21–5.89; *p* = 0.015). **Conclusions**: Circulating neopterin is independently associated with cardiovascular autonomic neuropathy in T2DM but not with classical microvascular complications. These findings suggest a potential role of immune-mediated mechanisms in the pathogenesis of diabetic cardiovascular autonomic neuropathy.

## 1. Introduction

Diabetes mellitus is a complex, chronic disease that requires continuous medical care and multifactorial risk-reduction strategies beyond glycemic control [1]. The global diabetes pandemic continues to expand at an alarming rate, with the prevalence of all types of diabetes nearly doubling from 4.7% in 1980 to 8.5% in 2014. It is estimated that approximately 700 million people worldwide will be living with diabetes by 2045, representing a one-third increase compared to current figures [2]. Chronic microvascular complications—including diabetic retinopathy, neuropathy, and nephropathy—as well as macrovascular complications such as myocardial infarction, cerebrovascular disease, and peripheral arterial disease remain major contributors to morbidity and mortality. Diabetic neuropathy develops in approximately 50% of patients during the course of the disease [3].

Diabetic polyneuropathy (DPN) represents one of the most common and clinically significant chronic complications of diabetes, affecting both the peripheral and autonomic nervous systems. It is defined as a heterogeneous group of neural disorders resulting from persistent hyperglycemia, metabolic imbalance, and microvascular dysfunction leading to structural and functional impairment of nerve fibers. Rather than a single entity, diabetic neuropathy comprises multiple clinical phenotypes ranging from asymptomatic sensory dysfunction to severe autonomic impairment with potentially life-threatening consequences [4].

Diabetic autonomic neuropathy (DAN) is a specific form of autonomic nervous system damage occurring in the presence of metabolic disturbances such as prediabetes or diabetes after exclusion of other causes. DAN results in impaired regulation of cardiovascular, gastrointestinal, genitourinary, and sudomotor functions. Unlike peripheral neuropathy, which often presents with sensory symptoms, autonomic neuropathy may remain subclinical in early stages while leading to severe systemic complications as the disease progresses [5].

Among the various forms of diabetic neuropathy, cardiovascular autonomic neuropathy (CAN) has particularly high prognostic significance. It is associated with abnormalities in cardiac autonomic regulation and increased risk of silent myocardial ischemia, arrhythmias, and sudden cardiac death [6]. Long-term follow-up studies have demonstrated increased overall mortality in patients with diabetes and confirmed CAN, with autonomic dysfunction acting as an independent predictor even after adjustment for traditional cardiometabolic risk factors [7]. Despite its clinical importance, DAN is frequently diagnosed at advanced stages when neural damage may already be irreversible. The absence of specific symptoms in early stages and the limited use of standardized diagnostic testing contribute to its underdiagnosis in routine clinical practice [8]. Consequently, delayed recognition increases the risk of complications, including gastroparesis, erectile dysfunction, bladder atony, hypoglycemia unawareness, and sudomotor dysfunction [4].

Within structured management strategies for diabetic complications, autonomic neuropathy often receives less attention compared with retinopathy or nephropathy. While screening for retinal disease and albuminuria is standardized, systematic screening for autonomic dysfunction is rarely performed despite existing guideline recommendations [4,7]. Emerging evidence suggests that autonomic dysfunction may develop even during the prediabetic stage, paralleling early metabolic and microvascular alterations [9].

Pathophysiology of Diabetic Neuropathy

The pathogenesis of DPN is multifactorial, and several studies indicate that pathogenic mechanisms differ between type 1 and type 2 diabetes. In type 2 diabetes, oxidative stress, vascular dysfunction, and metabolic disturbances play major roles, whereas in type 1 diabetes, chronic hyperglycemia acts as the primary trigger for multiple destructive molecular pathways. The main risk factors for DPN include chronic hyperglycemia, duration of diabetes and arterial hypertension. Additional contributors include obesity, smoking, and dietary exposure to advanced glycation end-products (AGEs) [10].

Despite substantial progress in understanding DPN pathophysiology, the underlying mechanisms remain incompletely elucidated. DPN is associated with hyperglycemia, dyslipidemia, insulin resistance, and protein catabolism. Hyperglycemia-induced oxidative stress plays a central role in peripheral nerve injury [11].

Inflammatory mechanisms further contribute to disease progression. Chronic low-grade inflammation promotes autonomic neuropathy, while autonomic dysfunction itself may enhance inflammatory responses, forming a bidirectional relationship [12]. Reduced vagal activity weakens the cholinergic anti-inflammatory pathway, whereas sympathetic overactivity stimulates macrophage activation and cytokine production [6,12,13,14]. These findings highlight the autonomic nervous system as an important regulator of immune and inflammatory processes [6,13,14,15].

Clinical Features of Cardiovascular Autonomic Neuropathy

Cardiovascular autonomic neuropathy remains one of the most studied yet underdiagnosed complications due to its subclinical early course [7,13,16,17]. Reduced heart rate variability (HRV) may be the earliest objective marker [7,12,18,19].

As the condition progresses, resting tachycardia (often up to 130 bpm), reduced exercise tolerance, and impaired chronotropic and inotropic responses may occur [7,13,19].

Orthostatic hypotension represents a late manifestation and is defined as a drop in systolic blood pressure ≥ 20 mmHg (or ≥30 mmHg in hypertensive patients) and/or diastolic blood pressure ≥ 10 mmHg within 3 min of standing [7,20].

Large cohort studies, including EURODIAB and Atherosclerosis Risk in Communities (ARIC), have demonstrated associations between autonomic dysfunction and increased risk of renal disease, retinopathy, and mortality [15,21,22,23,24,25,26].

Neopterin and Immune Activation

Neopterin is a biomarker of cellular immune activation produced primarily by macrophages and monocytes following stimulation by interferon-γ. Various cytokines and infectious stimuli—including interleukin-2 (IL-2), tumor-necrosis factor-α (TNF-α), lipopolysaccharide (LPS), and viral pathogens—can induce its production through activation of guanosine triphosphate cyclohydrolase I (GCH I) [27,28].

Neopterin is closely linked to the kynurenine pathway of tryptophan metabolism, a major metabolic route involved in NAD synthesis. Pro-inflammatory mediators activate enzymes such as indoleamine-2,3-dioxygenase, promoting conversion of tryptophan into kynurenine and other metabolites associated with metabolic and inflammatory disorders [29,30].

Neopterin production increases early during immune activation, preceding the peak of T-cell proliferation and antibody formation [31]. Elevated neopterin levels have been reported in multiple pathological conditions including cardiovascular disease, atherosclerosis [32], autoimmune disorders, chronic kidney disease, insulin resistance and diabetes [30]. Beyond its role as an inflammatory biomarker, neopterin also exhibits antioxidant properties and may inhibit NADPH oxidase activity, an important source of oxidative stress in neurological disorders [33,34].

The aim of the present study was to investigate whether circulating neopterin is associated with cardiovascular autonomic neuropathy in T2DM and whether this association is independent of traditional metabolic and renal risk factors.

## 2. Materials and Methods

### 2.1. Study Design

The study is cross-sectional and includes 129 participants (mean age 56.0 ± 9.2 years): 93 patients with type 2 diabetes and 36 individuals with obesity and normal glucose metabolism. The goal is to investigate the levels of neopterin and their relationship with cardiovascular autonomic neuropathy. Data collection was conducted over a one-year period.

The protocol of the study was in accordance with the Declaration of Helsinki and was approved by the Ethics Committee of the Medical University of Sofia (Protocol No. 11/11 July 2023). All participating subjects signed a written informed consent.

Inclusion criteria were age ≥ 18 years and diagnosis of type 2 diabetes mellitus according to ADA criteria or presence of obesity without carbohydrate metabolism disorders for the control group.

Exclusion criteria included acute inflammatory disease, active infection, malignancy, severe hepatic disease, end-stage renal disease, alcohol abuse, and other causes of peripheral or autonomic neuropathy.

### 2.2. Clinical and Anthropometric Assessment

All participants underwent a standardized clinical evaluation, including measurement of:

- Body weight (kg);
- Body mass index (BMI, kg/m^2^);
- Waist circumference (cm);
- Waist-to-hip ratio (WHR);
- Waist-to-stature ratio (WSR).

Blood pressure was measured after 10 min of rest using an automated sphygmomanometer.

Cardiovascular risk factors including arterial hypertension, dyslipidemia, smoking status, and metabolic syndrome were recorded.

### 2.3. Assessment of Diabetic Complications

Diabetic complications were evaluated using standardized clinical and instrumental methods.

Peripheral diabetic neuropathy was assessed using clinical examination and quantified by the Neuropathy Disability Score (NDS). The evaluation included assessment of tactile sensation using a 10 g monofilament applied at multiple standardized points on each foot, vibration perception using a 128 Hz Rydel–Seiffer tuning fork applied to the distal phalanx of the hallux, temperature sensation using a thermal discriminator, and tendon reflexes assessed with a neurological hammer. NDS over 5 (modified for lower limbs) was considered positive for peripheral neuropathy.

Peripheral nerve morphology was examined using corneal confocal microscopy (Heidelberg Retinal Tomograph III with Rostock Cornea Module, Heidelberg Engineering, Germany, Heidelberg). Standardized image acquisition protocols were applied, and quantitative analysis focused on: Corneal nerve fiber density (CNFD)—fibers per mm^2^; Corneal nerve branch density (CNBD)—branches per mm^2^; Corneal nerve fiber length (CNFL)—total fiber length per mm^2^. Images were analyzed using automated software (ACCMetrics, version 2.0). Diagnostic thresholds for corneal nerve parameters were defined according to the normative values established by Malik et al. [35].

Cardiovascular autonomic function was assessed using the Cardiosys Extra system (MDE GmbH, Heidelberg, Germany), which enables 12-lead ECG recording and analysis, including heart rate variability (HRV), QT interval variability, and performance of Ewing cardiovascular reflex tests. Standard Ewing tests were used to assess autonomic function, including: heart rate response to deep breathing; Valsalva maneuver; heart rate response to standing (30:15 ratio); blood pressure response to standing; blood pressure response to sustained handgrip. Heart rate variability was analyzed from a 5 min ECG recording using both time-domain and frequency-domain methods. Time-domain parameters included SDNN, RMSSD, pNN50, and HRV index. Frequency-domain analysis included high-frequency (HF) and low-frequency (LF) components, as well as the LF/HF ratio. Each test was scored as normal (0), borderline (1), or abnormal (2), and a composite autonomic score was calculated. Higher scores indicate more severe autonomic dysfunction. Based on the total score, patients were classified according to the Bellavere scale into absent, early, or definite autonomic neuropathy. Cardiovascular autonomic neuropathy was defined as the presence of at least two abnormal Ewing test results, in accordance with the Toronto Diabetic Neuropathy Expert Group criteria [19,36].

All assessments were performed in the morning after at least 12 h of fasting and abstinence from caffeine, alcohol, and medications affecting cardiovascular function, following a resting period of 15–20 min. Patients with conditions significantly interfering with autonomic testing were excluded where applicable.

Sudomotor function was evaluated using Sudoscan (Impeto Medical, Paris, France), a non-invasive method for assessing sweat gland function and small nerve fiber (C-fiber) integrity. Electrochemical skin conductance (ESC) was measured in microSiemens (µS), and an autonomic risk score was calculated to estimate the risk of autonomic neuropathy. Risk Stratification (CAN): Low risk: <25%; Moderate risk: 25–49%; High risk: ≥50% [37].

Diabetic nephropathy was defined as the presence of albuminuria and/or reduced eGFR.

Diabetic retinopathy was confirmed by ophthalmologic examination.

### 2.4. Laboratory Investigations

Fasting venous blood samples were collected after overnight fasting.

The following parameters were analyzed:

- Fasting plasma glucose
- Serum insulin
- HbA1c
- Lipid profile
- Serum creatinine
- Albumin-to-creatinine ratio

Normal glucose metabolism was defined according to ADA criteria (fasting glucose < 5.6 mmol/L, HbA1c < 5.7%, and/or normal OGTT where available).

Insulin resistance was estimated using the HOMA-IR index.

Neopterin concentrations were measured using a commercially available ELISA kit (IBL International GmbH, Hamburg, Germany) and expressed in nmol/L.

### 2.5. Statistical Analysis

The data were analyzed using the SPSS statistical software (version 23). Continuous variables are presented as mean ± standard deviation. Normality of data distribution was assessed using the Kolmogorov–Smirnov test. Variables with a normal distribution were analyzed using parametric methods, specifically analysis of variance (ANOVA). For variables that did not meet the assumptions of normality, non-parametric methods were applied. In particular, comparisons between two independent groups were performed using the Mann–Whitney U test. Correlation analyses were performed using Pearson correlation for normally distributed variables and Spearman’s rank correlation for non-normally distributed data. Multivariable logistic regression analysis was conducted to assess independent associations between variables. A *p*-value of less than 0.05 was considered statistically significant.

## 3. Results

### 3.1. Participants

The average age of the participants is 56.0 ± 9.2 years—93 patients with type 2 diabetes and 36 individuals with obesity and normal glucose metabolism.

The main characteristics of the patients are presented in Table 1.

Patients with T2DM and individuals with obesity had comparable body weight and BMI. However, subjects with T2DM demonstrated significantly higher markers of visceral adiposity, including: Waist circumference; Waist-to-hip ratio; Waist-to-stature ratio (*p* < 0.05).

### 3.2. Diabetes Characteristics and Treatment

For patients with T2DM, diabetes duration and antidiabetic treatment were documented. The mean duration of diabetes was 8.86 years. At the time of evaluation, 5.7% were without pharmacological therapy; 33% were receiving one antidiabetic drug; 29.5% were receiving two drugs; 19.3% were receiving three drugs; 10.2% were receiving four drugs; and 2.3% were receiving five antidiabetic drugs. The distribution of antidiabetic therapies included: Metformin—77.4%; Sulfonylureas—36.3%; DPP-4 inhibitors—12%; GLP-1 receptor agonists—20.7%; SGLT2 inhibitors—25.8%; and Insulin—21.7%.

### 3.3. Diabetes Complications

In the T2DM group, diabetic neuropathy was present in 72%, diabetic nephropathy in 23.7%, diabetic retinopathy in 14%, coronary artery disease in 18.7%, previous myocardial infarction in 10.9, stroke in 5.5%, and peripheral arterial disease in 5.5%.

### 3.4. Cardiometabolic Risk Profile

Cardiovascular risk factors are summarized in Table 2.

Patients with T2DM showed higher prevalence of arterial hypertension (91.4% vs. 52.8%, *p* < 0.05), higher prevalence of dyslipidemia (80.4% vs. 63.9%, *p* < 0.05) and higher prevalence of metabolic syndrome (92.2% vs. 50%, *p* < 0.05). A trend toward higher systolic blood pressure and triglycerides was observed in the diabetes group, while HDL cholesterol levels were significantly lower compared with the obesity group.

### 3.5. Neopterin Levels Between Study Groups

Circulating neopterin levels were comparable between patients with T2DM and individuals with obesity and normal glucose metabolism (6.05 ± 2.5 vs. 9.2 ± 11.4 nmol/L, *p* = 0.36), although a trend toward higher levels in the T2DM group was observed.

### 3.6. Neopterin and Diabetic Complications

No statistically significant differences in neopterin levels were observed in relation to peripheral diabetic neuropathy, diabetic nephropathy and diabetic retinopathy. These findings suggest that neopterin is not a sensitive biomarker for classical diabetic microvascular complications (Table 3).

**Figure 1:**
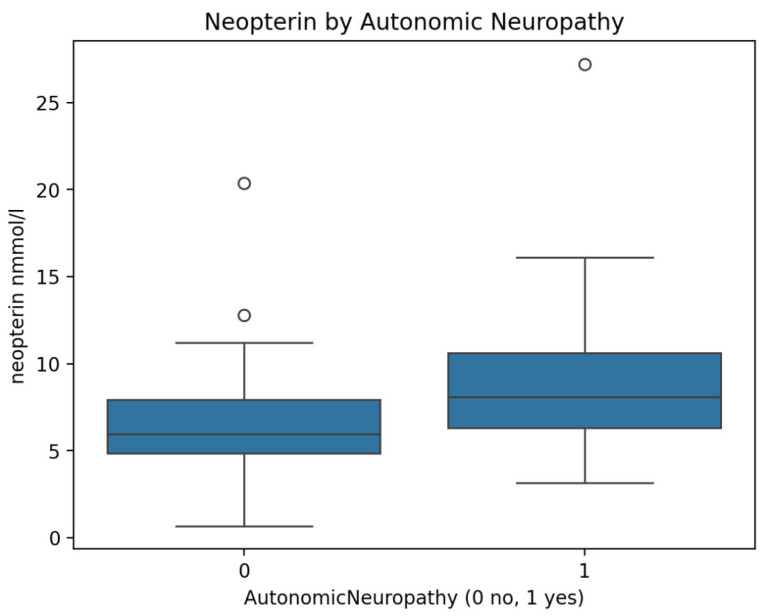
Neopterin concentrations in patients with and without autonomic neuropathy.

However, neopterin concentrations were significantly higher in patients with cardiovascular autonomic neuropathy compared with those without autonomic involvement (8.9 ± 3.9 vs. 6.7 ± 3.6 nmol/L, *p* = 0.013) (Figure 1).

### 3.7. Correlation Analyses

Circulating neopterin demonstrated significant correlations with markers of cardiovascular autonomic dysfunction—total autonomic neuropathy score (Ewing tests) (r = −0.233, *p* = 0.041) and sudomotor dysfunction score (r = −0.192, *p* = 0.034). Neopterin was also positively associated with metabolic parameters, including fasting plasma glucose (r = 0.523, *p* = 0.001), fasting insulin (r = 0.490, *p* = 0.002) and HOMA-IR (r = 0.666, *p* < 0.001). No significant correlations were observed with diabetes duration, visceral adiposity indices, blood pressure, lipid profile, HbA1c, albumin-to-creatinine ratio and corneal confocal microscopy parameters.

### 3.8. Neopterin and Renal Function

**Figure 2:**
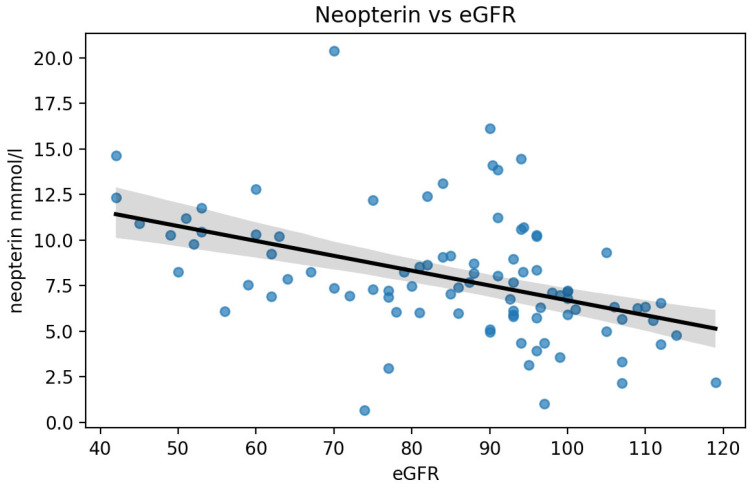
Neopterin levels versus eGFR.

**Figure 3:**
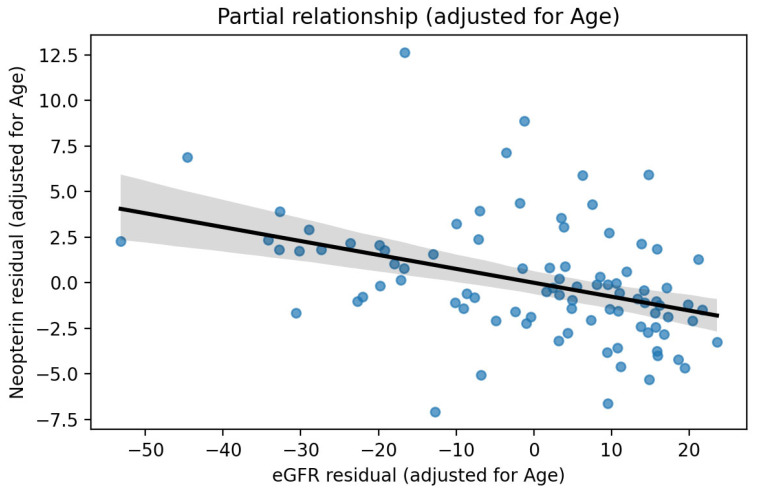
Relationship between neopterin levels and eGFR adjusted for age.

In the study cohort, circulating neopterin was inversely associated with kidney filtration. Specifically, neopterin showed a moderate negative monotonic correlation with estimated glomerular filtration rate (eGFR) (Spearman’s rho = −0.41, *p* < 0.001), indicating that higher neopterin concentrations tended to occur in participants with lower eGFR (Figure 2). This relationship remained significant after adjustment for age (Spearman’s rho = −0.39, *p* < 0.001) (Figure 3).

Consistent with this pattern, neopterin was positively correlated with serum creatinine (Spearman’s rho = 0.25, *p* = 0.0053). Neopterin was not significantly associated with albumin-to-creatinine ratio (ACR) in the available sample (Spearman’s rho = 0.10, *p* = 0.39, *n* = 80).

### 3.9. ROC Analysis

**Figure 4:**
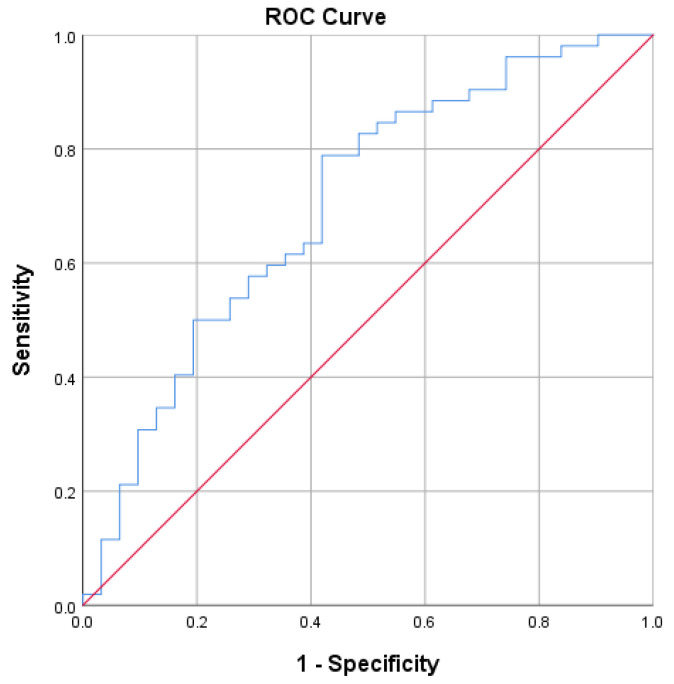
ROC curve for neopterin.

ROC analysis demonstrated moderate discriminatory ability of neopterin for cardiovascular autonomic neuropathy (Figure 4 and Table 4).

### 3.10. Logistic Regression Analysis

In univariable logistic regression analyses, higher circulating neopterin was associated with greater odds of cardiovascular autonomic neuropathy (OR 1.22, 95% CI 1.04–1.44, *p* = 0.017). After adjustment for age and sex, the association remained directionally consistent and statistically significant: each 1 nmol/L increase in neopterin was associated with a ~20% increase in the odds of cardiovascular autonomic neuropathy (OR 1.20, 95% CI 1.01–1.42, *p* = 0.038), indicating that the neopterin–cardiovascular autonomic neuropathy relationship was not explained by age or sex differences alone.

**Figure 5:**
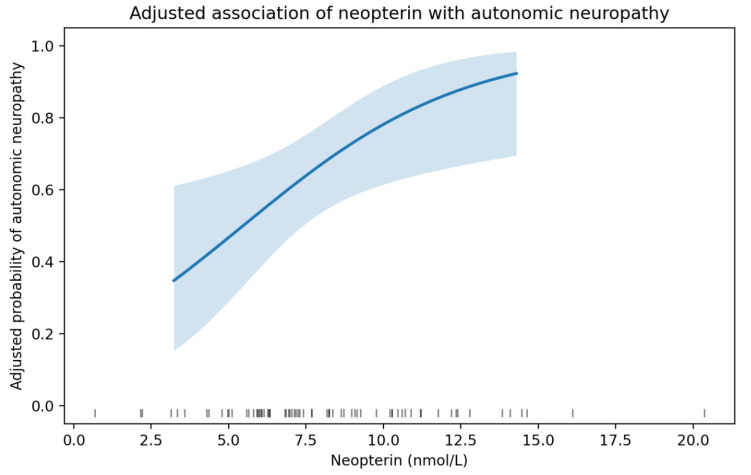
Adjusted association between neopterin and cardiovascular autonomic neuropathy. The figure displays the direction and magnitude of the association between neopterin (nmol/L) and the presence of cardiovascular autonomic neuropathy from a multivariable logistic regression model adjusted for age, HbA1c, BMI, eGFR and diabetes duration. Higher neopterin was associated with increased odds of cardiovascular autonomic neuropathy (adjusted OR 1.23 per 1 nmol/L, 95% CI 1.06–1.66; *p* = 0.015).

To examine whether circulating neopterin is independently associated with the presence of CAN, we performed a multivariable logistic regression analysis with cardiovascular autonomic neuropathy as the dependent variable (binary outcome). Neopterin was entered as the main exposure variable, and the model was adjusted for age, HbA1c, BMI, eGFR and diabetes duration. In adjusted logistic regression analysis (*n* = 70), higher neopterin levels were associated with greater odds of CAN after controlling for age, HbA1c, diabetes duration, BMI, and eGFR. Each 1-SD increase in neopterin (SD = 3.49 nmol/L) was associated with a 2.67-fold higher odds of CAN (adjusted OR 2.67, 95% CI 1.21–5.89; *p* = 0.015), while each 1 nmol/L increase in its levels was associated with 1.32-fold higher odds of CAN neuropathy (adjusted OR 1.32, 95% CI 1.06–1.66; *p* = 0.015). The adjusted association is illustrated by the model-based predicted probability of cardiovascular autonomic neuropathy across neopterin concentrations (Table 5 and Figure 5).

In the same model, age (*p* = 0.508), HbA1c (*p* = 0.160), and BMI (*p* = 0.508) were not significantly associated with CAN. Diabetes duration was also not statistically significant in the adjusted model.

The reduced sample size (*n* = 70) in the multivariable model was due to missing data for some covariates (e.g., eGFR, HOMA-IR, or complete autonomic assessment), and only participants with type 2 diabetes and complete data were included.

## 4. Discussion

The present study demonstrates that circulating neopterin is independently associated with cardiovascular autonomic neuropathy in patients with type 2 diabetes mellitus, while no significant relationship was observed with classical diabetic microvascular complications such as peripheral neuropathy, nephropathy, or retinopathy. These findings suggest that immune-mediated inflammatory mechanisms may play a more prominent role in the development of autonomic dysfunction than in traditional microangiopathic complications of diabetes.

**Neopterin as a Marker of Immune Activation in Type 2 Diabetes**

Neopterin is a well-established biomarker of cellular immune activation, produced by macrophages upon stimulation by interferon-γ released from activated T-helper 1 lymphocytes [33]. Elevated circulating neopterin levels reflect activation of the Th1 immune pathway and increased oxidative stress, both of which are known to contribute to metabolic and vascular pathology [38,39,40]. Chronic low-grade inflammation is a central component of T2DM pathogenesis and is strongly associated with insulin resistance and endothelial dysfunction [41].

Previous studies have demonstrated increased neopterin concentrations in metabolic disturbances and cardiovascular disease, supporting its role as a marker of systemic inflammatory activation [42,43]. However, data regarding its association with diabetic neuropathy remain limited, particularly for autonomic neuropathy. Our results extend current knowledge by showing that neopterin is selectively associated with cardiovascular autonomic dysfunction rather than with classical microvascular complications. Although peripheral neuropathy was primarily assessed clinically, corneal confocal microscopy was also performed, providing an objective and sensitive measure of small fiber integrity. The lack of association between neopterin and corneal nerve parameters further supports the specificity of the observed relationship with CAN.

Interestingly, in the present study, neopterin levels were not significantly different between individuals with T2DM and those with obesity and normal glucose metabolism, although a trend toward higher values in the diabetes group was observed. This finding should be interpreted with caution, as obesity itself is characterized by chronic low-grade inflammation and increased macrophage activation, which may already lead to elevated neopterin levels. Therefore, the absence of a significant difference between groups may reflect shared inflammatory pathways rather than the absence of diabetes-specific effects. This observation further supports the concept that inflammatory mechanisms linking metabolic dysfunction and neural damage may be present already at the stage of obesity and insulin resistance, prior to overt hyperglycemia [44].

**Association Between Neopterin and Cardiovascular Autonomic Neuropathy**

One of the most important findings of the present study is the significant association between circulating neopterin and CAN. Patients with autonomic dysfunction demonstrated significantly higher neopterin levels compared with those without autonomic involvement. Moreover, neopterin showed significant correlations with both the Ewing autonomic score and sudomotor dysfunction, suggesting that inflammatory activation may contribute to both parasympathetic and sympathetic fiber impairment.

Cardiovascular autonomic neuropathy is a serious and frequently underdiagnosed complication of diabetes, associated with increased risk of arrhythmias, silent myocardial ischemia, and mortality [5,8]. Although chronic hyperglycemia has traditionally been considered the primary pathogenic factor, emerging evidence indicates that inflammation and oxidative stress also play critical roles in neural injury [45]. In this context, hyperglycemia-induced metabolic disturbances are known to promote oxidative stress and mitochondrial dysfunction, contributing to neuronal injury and impaired autonomic function. These processes, together with inflammation-mediated damage, may play a role in the structural and functional alterations of autonomic nerve fibers observed in CAN [11,46,47].

Autonomic fibers, particularly small unmyelinated fibers, appear to be especially vulnerable to metabolic and inflammatory injury [48]. Macrophage activation and cytokine-mediated neuroinflammation may contribute to mitochondrial dysfunction, axonal degeneration, and impaired neural repair mechanisms [49,50,51]. Neopterin, as a marker of macrophage activation and reactive oxygen species formation, may therefore reflect these pathophysiological processes [38,39].

Importantly, the association between neopterin and cardiovascular autonomic neuropathy remained statistically significant after adjustment for age, HbA1c, BMI, renal function, and diabetes duration. This suggests that inflammatory activation may represent an independent pathogenic pathway beyond traditional metabolic risk factors.

**Relationship Between Neopterin and Insulin Resistance**

An additional important observation is the significant correlation between circulating neopterin and markers of insulin resistance, including fasting insulin and HOMA-IR. This relationship further supports the link between immune activation and metabolic dysfunction [52].

Insulin resistance is increasingly recognized as an inflammatory condition characterized by macrophage infiltration in adipose tissue and increased production of pro-inflammatory cytokines [53,54]. Interferon-γ signaling and macrophage polarization toward a pro-inflammatory phenotype may contribute to both systemic inflammation and neural damage [55,56]. Interestingly, neopterin did not correlate with HbA1c in the present study, suggesting that inflammatory activation may be more closely related to insulin resistance than to chronic glycemic exposure.

This observation aligns with emerging data indicating that insulin resistance may be a stronger predictor of neuropathy progression than HbA1c in certain patient populations.

**Neopterin and Renal Function**

The present study also demonstrated a moderate inverse association between neopterin and eGFR, as well as a positive association with serum creatinine. These findings are consistent with previous reports showing that neopterin accumulates in patients with reduced renal clearance [57,58]. Importantly, the association between neopterin and cardiovascular autonomic neuropathy remained significant after adjustment for eGFR, indicating that renal function does not fully explain the observed relationship. However, residual confounding cannot be fully excluded. Neopterin is partly cleared by the kidneys, and reduced renal function may contribute to its accumulation [57]. In addition, chronic kidney disease is associated with increased systemic inflammation, which may further influence circulating neopterin levels. The nonlinear nature of the association between neopterin and eGFR suggests that renal elimination may influence circulating levels, while inflammatory activation likely represents an additional contributing mechanism.

**Predictive Value of Neopterin for Cardiovascular Autonomic Neuropathy**

ROC analysis demonstrated moderate discriminatory performance of neopterin for detecting cardiovascular autonomic neuropathy. Although not sufficient as a standalone diagnostic marker, this finding suggests potential utility for risk stratification.

Moreover, multivariable logistic regression analysis showed that each standard deviation increase in neopterin was associated with approximately a 2.7-fold higher odds of CAN. This relatively strong association highlights the potential clinical relevance of inflammatory biomarkers in identifying patients at increased risk.

Given the often subclinical nature of early autonomic dysfunction, biomarkers reflecting underlying pathogenic mechanisms may improve early detection strategies.

**Study limitations**

This study has several limitations. First, its cross-sectional design does not allow causal inference. Second, the sample size is relatively modest, particularly for multivariable analyses. Third, some measurements were not available for all participants, which reduced the sample size in adjusted models. Finally, autonomic neuropathy assessment was primarily based on cardiovascular reflex tests, and more advanced techniques (e.g., HRV spectral analysis or imaging methods) were not included.

## 5. Conclusions

The present study demonstrates that circulating neopterin is independently associated with autonomic neuropathy in patients with type 2 diabetes mellitus, while no significant relationship was observed with classical diabetic microvascular complications such as peripheral neuropathy, nephropathy, or retinopathy. These findings suggest that immune-mediated inflammatory mechanisms may play a more prominent role in the development of autonomic dysfunction than in traditional microangiopathic complications.

Circulating neopterin was also associated with markers of insulin resistance and renal function, supporting the concept that systemic inflammation contributes to metabolic and neurodegenerative pathways in diabetes. Importantly, the association between neopterin and autonomic neuropathy remained significant after adjustment for major metabolic and renal confounders, indicating an independent relationship.

Although the predictive performance of neopterin was moderate, its association with autonomic dysfunction highlights the potential clinical value of inflammatory biomarkers for early risk stratification.

Further prospective studies are required to determine whether neopterin may serve as a predictive biomarker for the progression of diabetic cardiovascular autonomic neuropathy and to clarify the underlying immunometabolic mechanisms.

## Figures and Tables

**Table 1:** Anthropometric characteristics of the study population.

	Group 1 Diabetes Type 2	Group 2 Obesity and Normal Glucose Metabolism
Weight (kg)	98.2 ± 17.9	94.6 ± 16.3
BMI (kg/m^2^)	34.9 ± 5.8	35.2 ± 3.8
Waist circumference (cm)	112.3 ± 14.2 *	105.0 ± 10.9
WSR	0.76 ± 0.07 *	0.63 ± 0.05
WHR	0.98 ± 0.11 *	0.90 ± 0.08

* *p* < 0.05.

**Table 2:** Cardiovascular risk factors.

	Group 1 Diabetes Type 2	Group 2 Obesity and Normal Glucose Metabolism
Systolic blood pressure	133.0 ± 16.3	128.11 ± 12.1
Diastolic blood pressure	80.9 ± 9.01	81.0 ± 9.4
Prevalence of arterial hypertension	91.4% *	52.8%
Total cholesterol	5.3 ± 1.5	5.4 ± 1.1
LDL-cholesterol	3.0 ± 1.1	3.4 ± 0.9
HDL-cholesterol	1.2 ± 0.3 *	1.4 ± 0.3
Triglycerides	2.6 ± 2.7	1.7 ± 1.1
Prevalence of dyslipidemia	80.4% *	63.9%
Prevalence of smoking	39.8%	50%
Metabolic syndrome	92.2% *	50%

* *p* < 0.05.

**Table 3:** Neopterin levels according to diabetic complications.

Complication	Status	Neopterin (nmol/L), Mean ± SD
Diabetic neuropathy	No	8.13 ± 13.92
	Yes	8.54 ± 3.26
Diabetic nephropathy	No	8.36 ± 10.77
	Yes	8.29 ± 2.73
Diabetic retinopathy	No	8.27 ± 10.29
	Yes	8.99 ± 4.48

**Table 4:** Area Under the Curve (AUC) Analysis for Neopterin.

Area Under the Curve				
Test Result Variable(s):			Neopterin	
Area	Std. Error ^a^	Asymptotic Sig. ^b^	Asymptotic 95% Confidence Interval	
			Lower Bound	Upper Bound
0.702	0.060	0.002	0.584	0.821

^a^. Under the nonparametric assumption. ^b^. Null hypothesis: true area = 0.5.

**Table 5:** Multivariable logistic regression for presence of cardiovascular autonomic neuropathy.

Predictor	Adjusted OR (95% CI)	*p*-Value
Neopterin (per 1 SD)	2.67 (1.21–5.89)	0.015
Neopterin (per 1 nmol/L)	1.32 (1.06–1.66)	0.015
Age (year)	0.99 (0.93–1.07)	0.508
HbA1c (%)	0.69 (0.42–1.13)	0.160
BMI (kg/m^2^)	1.08 (0.98–1.19)	0.508
eGFR (mL/min/1.73 m^2^)	1.03 (0.99–1.07)	0.129
Diabetes duration (years)	1.1 (0.97–1.24)	0.14

## Data Availability

The data presented in this study are available on request from the corresponding author. The reason for restriction due to privacy or ethical restrictions.

## Abbreviations

The following abbreviations are used in this manuscript:

ACR	Albumin-to-creatinine ratio
ADA	American Diabetes Association
AGEs	Advanced glycation end-products
AN	Autonomic neuropathy
ARIC	Atherosclerosis Risk in Communities study
ATP	Adenosine triphosphate
BMI	Body mass index
BP	Blood pressure
CAN	Cardiovascular autonomic neuropathy
DAN	Diabetic autonomic neuropathy
DBP	Diastolic blood pressure
DM	Diabetes mellitus
DPN	Diabetic polyneuropathy
DR	Diabetic retinopathy
ELISA	Enzyme-linked immunosorbent assay
eGFR	Estimated glomerular filtration rate
GM-CSF	Granulocyte-macrophage colony-stimulating factor
GAPDH	Glyceraldehyde-3-phosphate dehydrogenase
GCH I	Guanosine triphosphate cyclohydrolase I
HbA1c	Glycated hemoglobin
HDL	High-density lipoprotein
HOMA-IR	Homeostasis model assessment of insulin resistance
HRV	Heart rate variability
IFN-γ	Interferon gamma
IL-2	Interleukin 2
IR	Insulin resistance
LDL	Low-density lipoprotein
LPS	Lipopolysaccharide
NAD	Nicotinamide adenine dinucleotide
NADPH	Nicotinamide adenine dinucleotide phosphate
NF-κB	Nuclear factor kappa B
NO	Nitric oxide
NOX	NADPH oxidase
OGTT	Oral glucose tolerance test
OR	Odds ration
PARP	Poly(ADP-ribose) polymerase
PI3K	Phosphoinositide 3-kinase
PKC	Protein kinase C
ROS	Reactive oxygen species
SBP	Systolic blood pressure
SD	Standard deviation
SGLT-2	Sodium-glucose cotransporter 2
T2DM	Type 2 diabetes mellitus
TNF-α	Tumor necrosis factor alpha
WHR	Waist-to-hip ratio
WSR	Waist-to-stature ratio
